# Conical Refraction Bottle Beams for Entrapment of Absorbing Droplets

**DOI:** 10.1038/s41598-018-23399-y

**Published:** 2018-03-22

**Authors:** Michael Esseling, Christina Alpmann, Jens Schnelle, Robert Meissner, Cornelia Denz

**Affiliations:** Institute of Applied Physics, University of Muenster, 48149 Muenster, Germany

## Abstract

Conical refraction (CR) optical bottle beams for photophoretic trapping of airborne absorbing droplets are introduced and experimentally demonstrated. CR describes the circular split-up of unpolarised light propagating along an optical axis in a biaxial crystal. The diverging and converging cones lend themselves to the construction of optical bottle beams with flexible entry points. The interaction of single inkjet droplets with an open or partly open bottle beam is shown implementing high-speed video microscopy in a dual-view configuration. Perpendicular image planes are visualized on a single camera chip to characterize the integral three-dimensional movement dynamics of droplets. We demonstrate how a partly opened optical bottle transversely confines liquid objects. Furthermore we observe and analyse transverse oscillations of absorbing droplets as they hit the inner walls and simultaneously measure both transverse and axial velocity components.

## Introduction

The versatility of optical techniques has boosted ever-increasing interest in their exploitation for micromechanical purposes. Focused light fields can be accurately controlled and rapidly changed with phase modulation techniques^1^, and their ability to manipulate matter has been demonstrated already in the 1970s^2^. Forces in the femto - to piconewton range can be exerted directly by optical tweezers^3^, holographic optical tweezers^4^, or modulated potential landscapes^5^, all of which are able to confine transparent objects in high intensity regions, provided that their refractive index is higher than that of its surrounding. Recently, some indirect techniques were proposed, in which light does not directly interact with the objects under investigation, but its influence is mediated by thermal or electric media^6–9^. These secondary techniques, such as *photophoresis* or *dielectrophoresis*, contribute to the tool-set of optical confinement, a method to handle also non-transparent particles, which is impossible in direct optical tweezers due to excessive scattering forces. Among these techniques, photophoresis has been demonstrated as a versatile mechanism to handle highly-absorbing airborne particles. In this paper, we demonstrate the application of conical refraction to the spatial confinement of absorbing airborne droplets, thereby not only extending the optical trapping toolset for absorbing particles, but supply a highly flexible, versatile trapping means that paves the way to trap absorbing particles with the same ease as dielectric transparent particles. Although the effect of conical refraction has been first predicted by Hamilton and shortly after been experimentally observed by Lloyd in 1832^10^, it was not until the beginning of this century that its importance was rediscovered (see refs^11,12^ and references therein) as a powerful tool for advanced trapping purposes down to single atom and Bose-Einstein condensate trapping^13^. CR describes the circular split-up of light propagating along the optical axis of a biaxial crystal and hence lends itself to the construction of circular-symmetrical optical traps. One of the biggest advantages of this trap for absorbing particles compared to other methods for bottle beams^8^ is its feature to be opened and closed in a defined way by tuning the polarisation states of the conical rings. We investigate different protocols to fill this trap and present a sophisticated dual-view observation technique to locate the droplet position in three dimensions.

## Methods

### Photophoretic manipulation of absorbing droplets

Photophoresis, which can also be described as *light-induced thermophoresis*, describes the migration of microscopic particles in a temperature-gradient that is generated by inhomogeneous heating of the particle surface by light^14^, an effect that obviously comes into play the more absorbing a particle is. A resulting net force on the particle originates from the transfer of momentum from the hot particle surface to the surrounding medium. Although moderately absorbing particles may experience *negative photophoresis*, which drags objects towards the source of light, thereby realizing a versatile technique of a tractor beam without demanding modifications of other tractor beam techniques^15,16^, most implementations exploit *positive photophoresis*, which is the dominant effect for highly absorbing media and pushes particles in the axial propagation direction. For details about this effect, the reader is referred to ref.^17^ and references therein.

In order to confine absorbing matter, it is necessary to construct a hollow light field with high intensity regions, which is subsequently able to trap and guide absorbing particles. This represents a static hollow beam^18,19^. Since the first proof-of-principle demonstrations in laser beam shaping and atom trapping^20–22^, considerable advances in tailored beam shaping have led to the development of dynamic traps^7,8^ and allowed extending secondary optical trapping from absorbing solids to absorbing liquids^23^. Trapping absorbing liquids imposes a number of challenges on optical trapping such as convection and premature evaporation. In a first realization, it could be shown that airborne inkjet droplets can experience multiple interaction with a focused sheet of light. In a second step, this milestone allowed estimating the threshold intensity for photophoresis with droplets^23^. Finally, in a third step, it was found that the forces acting on an absorbing droplet can be decomposed in a transverse and an axial component. During photophoretic interaction, the absorbing droplet (partly) enters the focused light field, hence induces its own “shadow”, and thus an axial photophoretic force is exerted in the direction of light propagation (cf. also ref.^23^). As a consequence, a photophoretic trap must be able to balance the axial force components to confine the droplet in all three dimensions. This prerequisite is necessary: on the one hand, to characterize droplet lifetime, evaporation rate as well as possible elastic deformations during the impact on the light field; and at the other hand to apply the approach to advanced optical trapping.

### Conical refraction optical bottle

The construction of a three-dimensional droplet trap based on the concepts of photophoresis requires combining concepts from different fields of optical manipulation: On the one hand, it has been shown that ink droplets can be stopped and guided by focused laser light^23^, an indispensable ingredient to optical manipulation. On the other hand, confinement of a droplet can be realized by an *optical bottle beam*. Thus, a combination of both discoveries allow for 3D confinement^8^. The remaining challenge is that the previously generated optical bottles–e.g. consisting of axially displaced vortices^8^–are of perfect circular symmetry. As a result, they are fully closed, and the photophoretic high intensity barrier keeps absorbing particles outside as much as it retains them inside, once they are trapped. A straight forward approach to overcome this challenge is a statistical approach flooding an optical hollow trap with a high number of particles in such a way that eventually a single one stays inside the trap. This approach, however, cannot be transferred to single droplets. Eventually, a large number of droplets may coalesce to volumes that are too large to fit into a hollow trap.

**Figure 1:**
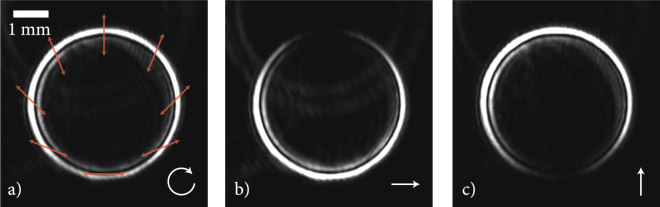
Generation of optical bottle beams by conical refraction: (**a**) Circular split-up of different polarisation angles of a circularly polarised beam, (**b**) opening the top and (**c**) bottom of the bottle beam by changing the polarisation state of the incident light field (indicated by white arrows). The diameter of the CR ring needs to be adapted to the droplet size (compare Fig. 3).

**Figure 2:**
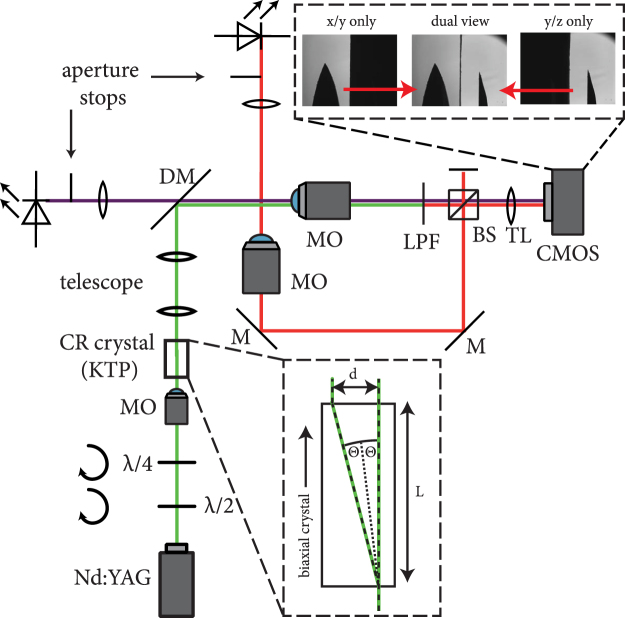
Setup for the generation of conical refraction bottle beams and their observation: A frequency-doubled Nd:YAG laser whose polarisation is adjusted by a combination of half-wave plate and quarter-wave plate, is focused onto a KTP crystal. The resulting bottle beam is relayed and demagnified by a telescope to the observation volume, where its interaction with absorbing droplets is imaged by a dual view optical setup for x/y and y/z planes, respectively. Upper inset shows an exemplary view of the dual view camera during adjustment with a needle tip. Lower inset illustrates the circular split-up during *internal conical refraction*. For details about abbreviations refer to text.

An intriguing assay for infiltrating liquid matter inside a photophoretic trap has been demonstrated by Turpin *et al*.^24^. The authors have exploited the effect of conical refraction (CR) to construct a flexible bottle beam. What makes CR particularly interesting for trapping purposes is the fact that the azimuthal intensity distribution of the ring-shaped light structure is determined by the orientation of the polarisation vector with respect to the crystal axes. Figure 1 shows the polarisation structure of a ring generated by conical refraction. Unpolarized or circularly polarized light, for which no particular static polarisation angle can be fixed, experiences a circular split-up while perpendicular linear polarisations allow to open the top or the bottom of the circle. In more detail, Hamilton’s early investigations already revealed two different phenomena: *internal conical refraction* and *external conical refraction*. Internal CR takes place only for infinitely narrow yet parallel rays of light incident along the optical axis. These rays will then be split up along a cone, as visualized in the sketch of Fig. 2, and exit the crystal in a circle, whose diameter *d* can be calculated by the apex angle and the length of the crystal^25^:

1 $$d=2L\,\sin \,{\rm{\Theta }}\approx 2L{\rm{\Theta }}$$

where the apex angle is determined by the optical properties of the crystal via ${\rm{\Theta }}\mathrm{=1/}{n}_{2}\sqrt{({n}_{2}-{n}_{1})({n}_{3}-{n}_{2})}$^25^. For KTP (*n*_1_ = 1.7779, *n*_2_ = 1.7887, *n*_3_ = 1.8887), which is used in the current study, this apex angle is Θ = 0.0184 rad = 1.053°, so the assumption of small angles is well-justified. The main indices of refraction *n*_*i*_ are identified by the half of the main axes of a triaxial ellipsoid (index ellipsoid) which can be derived from the inverse dielectric tensor. On the other hand, for a beam that is focused onto the surface of the CR crystal in a way that all refracted rays inside the crystal propagate along the optical axis, external conical refraction takes place, i.e. the beams exit in an opening cone. In experiments, a combination of both effects exists, as discussed by Sokolovskii *et al*.^26^. The overlap of the opening cone of internal CR and the converging cone of external CR is at the origin of the concept to exploit conical refraction to create polarisation-dependent variable bottle beams, and allows for the efficient construction of a true 3D photophoretic trap.

## Results

### Dual view observation setup of droplet entrapment

**Figure 3:**
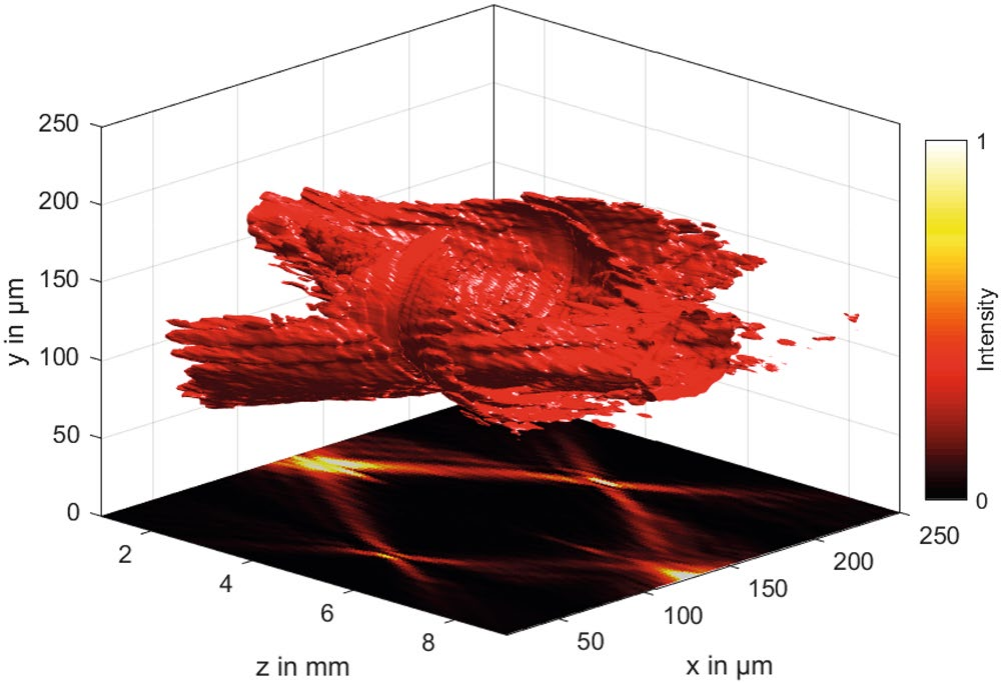
A 3D look inside an experimentally realized optical bottle beam. The opening of the bottle is easily facilitated by changing the polarisation from circular to linear, where the angle of the linear polarisation influences the azimuthal position of the opening. The projection of the lateral cut through the center of the beam indicates the well-focused high intensity regions at both ends of the bottle beam as well as in the focus plane, where the two CR rings are sharply observable. In between, the intensity gradient is less steep, effectively reducing the photophoretic force.

Conically refracted beams have been generated, characterized and analyzed with the setup shown in Fig. 2. To generate the modulated beam, a frequency-doubled Nd:YAG laser (*λ* = 532 nm) passes through motorized *λ*/2 and *λ*/4 plates. Both wave plates can be independently rotated to achieve all polarisation states from linear, elliptical to perfectly circular polarisation by choosing an appropriate orientation of the *λ*/2 plate relative to the orientation of the fast axis of the *λ*/4 plate. The light is focused onto the surface of a KTP crystal by a 4x microscope objective (MO) (Nikon, NA 0.1). The convergence angle of the focused light of 5.7° is much larger than the angle of internal CR of Θ = 1.053°, so a mixture of internal and external conical refraction will lead to the dual-cone interference as described above. The size of the hollow region is fixed by the optical properties of the KTP crystal and its geometrical size. With a length of *L* = 10 mm, the diameter of the ring induced by internal CR can be estimated to d_internal_ = 2⋅10⋅0.0184 = 368 μm. By a flexible telescope optical system, the diameter can be adapted to the requirements of the respective droplet experiment. A three-dimensional isosurface scan through a CR optical bottle beam is shown in Fig. 3. It was obtained by scanning the movable microscope objective of Fig. 2 in the axial direction. The plot clearly shows the two intersecting cones as described in ref.^26^. To demonstrate the versatility of CR optical bottle beams, the polarisation of the beam was adjusted parallel to the optical table, so that the top opening allows looking inside the trap. The bottle beam intensity is well focused at its end points and in the focal plane, where two bright rings are separated by the Poggendorff dark ring^27^. In the areas in between, the intensity is less concentrated, it is illustrated in the projection on the bottom of the plot. These areas of blurred intensity are of importance for optical trapping efficiency. By comparison of d_internal_ with the experimental results of Fig. 1, we find that in the experiment the diameter of the CR ring is significantly larger confirming that not only internal, but also external CR occurs.

To demonstrate photophoretic manipulation by conical refraction traps, single airborne droplets are generated by a printer cartridge with a fixed volume and ink concentration as reported in ref.^23^ These droplets are shot upright against gravity, and are detected on their way down. During their descent before any interaction with the light field, the liquid objects reliably reach a constant terminal velocity *v*_y-terminal_ ≈ −70 mms^−1^, which is comparably lower than the exit velocity *v*_y-exit_ ≈ 2 ms^−1^. Nevertheless this method is far more reliable than measuring the velocity at the turning point of the trajectory which may vary due to air fluctuations. Since the size of the droplets is rather mono-disperse with a mean diameter of 50 μm, the optical bottle beam is demagnified to provide the desired spatial confinement. Therefore, it is relayed to the desired interaction volume by a telescope, which demagnifies the CR beam by a factor of approximately 3.5. Since a defined trapping volume is most sufficient to confine droplets with small deviations in size, on the one hand the bottle beam has to be adjusted to match droplets with smaller or bigger volumes. On the other hand, this selectivity might be used to implement sorting of poly-disperse droplets by size. In this context we propose combination with the photophoretic trampoline, which also allows a size selective sorting, to enlarge the variety of possible trapping and sorting scenarios.

In previous approaches to implement optical bottle beams, tracking in 3D was achieved by employing two distinct cameras for the front/side observation^8^. Here, we use a single high-speed camera (CMOS in Fig. 2, *Imaging Solutions NX8-S2*, 4000 full fps) for the spatio-temporal recording of the droplet movement by dividing the large sensor into a dual-view configuration similar to the one presented in ref.^28^ Thus, we take advantage of the fact that ideally, the droplet is spatially confined, so only a very small fraction of the field-of-view needs to be used. The recorded high speed videos were analysed by freely available Center of Mass particle tracking algorithms further improved to yield maximum tracking accuracy and optimum particle linking^29,30^. Tracked positions were further evaluated for velocity. The dual-view setup consists of two LEDs which illuminate the transverse planes of the interaction volume, respectively. Their image is captured by two infinity-corrected microscope objectives (MO) and combined in a 50/50 beam-splitter (BS). The use of infinity-corrected MOs allows the use of the same 200 mm tube lens to form the image. To avoid poor contrast and bad illumination scenarios from the overlay of the two images, razorblades are imaged into the focal planes of the MOs, so that each side of the camera is only illuminated by light from its corresponding plane (cf. upper inset in Fig. 2).

### Droplet oscillations in a photophoretic optical bottle

**Figure 4:**
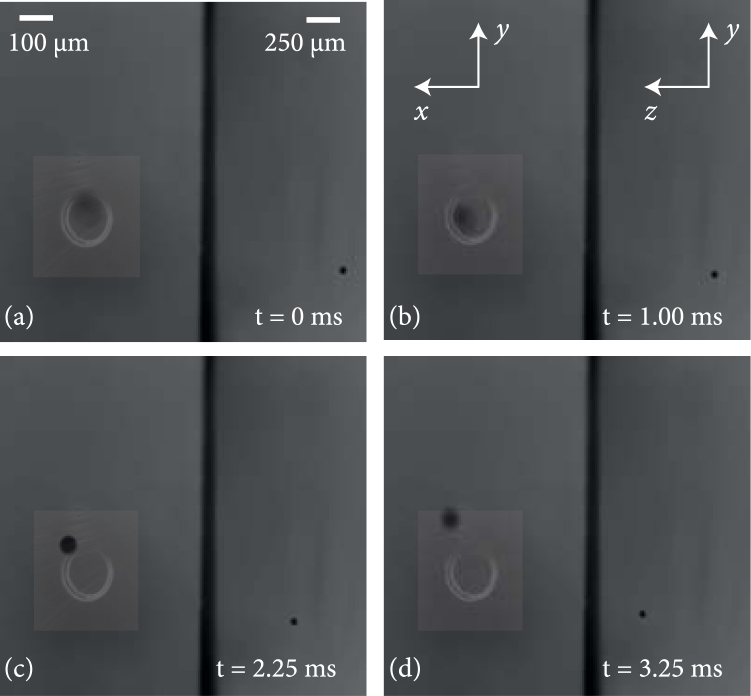
Result of droplet interaction with a bottle beam, whose top section was fully opened (cf. also Fig. 3). The droplet is stopped by photophoretic forces, but due to the previously observed bounces, it leaves the optical trap again.

The optical trap is filled by catapulting a single optical droplet upwards so that on its way down it passes directly through the CR bottle beam. The laser light, which is incident on the CR crystal was adjusted to be linearly polarized so that the top section of the bottle beam is fully open (cf. also Fig. 1). Figure 4 shows the result of the interaction of an inkjet droplet falling at its terminal velocity. As in the previous experiments with the *optical trampoline*, the droplet experiences a strong restoring force which lets it bounce on the trampoline, or–as in this case–on the optical bottle beam and out of the trap (cf. also the supplementary videos). Since the top of the bottle is open, the droplet is free to exit again.

**Figure 5:**
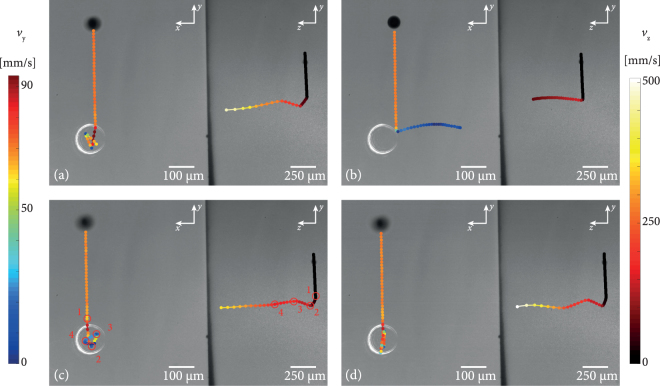
Droplet interaction with partly opened CR bottle beam (intensity ratio top/bottom: 0.7/1): In contrast to the fully opened bottle beam in Fig. 4, the droplet does not exit the photophoretic trap any more as depicted with the trajectory lines. While the transverse confinement is significantly improved, there is still a great momentum transfer in the axial direction through photophoretic interactions between the droplet and the bottle beam walls. The resulting velocity is illustrated by the color coding of each trajectory - *v*_*y*_ for the left site and *v*_*z*_ for the right side of each subfigure. Please note that the opening of the bottle on the right side is an artefact from a polarisation sensitive longpass filter used to block the strong trapping beam.

To prevent the droplet from leaving the optical trap in any lateral direction, the bottle can be partly opened: By adjusting the polarisation from linear to elliptical, the intensity ratio between top and bottom section can be continuously tuned. In our experiments, a value of 0.7/1 was found to be the optimal compromise: The droplet is fast enough to enter the trap, but is stopped by the bottom of high intensity light. On the rebound, it is slower than on its way down (measured as *v*_*y*_ ≈ 50 mms^−1^ by video microscopy as compared to *v*_y − terminal_ ≈ −70 mms^−1^ on the descent), so even the reduced power of the upper border of the trap is sufficient to keep the liquid droplet inside, as shown in Fig. 5. In these images, the opening of the bottle beam with the reduced intensity ratio points to the top. In order to demonstrate the direct correlation between droplet movement and trap position, a longpass filter blocks out the high-intensity 532 nm light almost entirely to avoid overexposure of the camera. As a side effect, the polarisation-dependence of the filter generates the impression that the bottle opening points to the side.

Note that a third possible solution to keep the droplet in the trap is to close it after the droplet has entered, e.g. by a dynamic polarisation switching device. Because the time from the detection of a droplet impact until the final closing of the trap must not be longer than approximately 500 μs, this technique can be implemented by using standard driving electronics combined with a Pockels cell.

**Figure 6:**
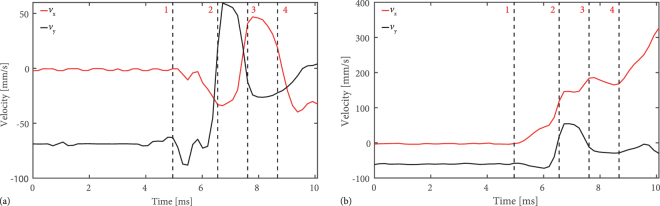
Evolution of droplet velocity over time taken from Fig. 5(c) caused by interactions with the bottle beam walls. The left graph is related to the x/y-view and right graph illustrates the velocity evolution in the y/z-view of Fig. 5(c). For each interaction inside the bottle beam, numbers are used to get a relation between position (Fig. 5(c)), time and the velocity (Fig. 6).

**Figure 7:**
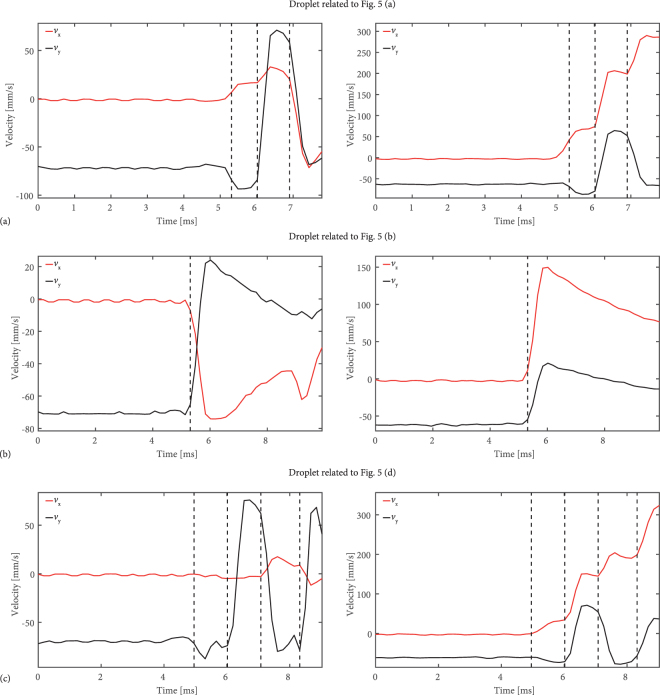
Evolution of droplet velocity over time for droplets (**a**), (**b**) and (**d**) in Fig. 5 to illustrate the results for different scenarios. In all three cases the droplet reaches the trapping volume at a different position (compare left hand side of each subfigure in Fig. 5. Excepting Fig. 5(b) - corresponds to (**b**) in this figure - several interaction can be observed by their change in velocity in *v*_*x*_, *v*_*y*_ and *v*_*z*_, which demonstrates a robust transverse confinement.

The supplementary dual view videos as well as the results presented in Fig. 5 show that the transverse confinement is sufficient and oscillations between the bottle beam walls can be observed for four different droplets (a–d). As it can be seen, the resulting trajectory is highly depending on the initial path down to the photophoretic trap, since there is only a small area allowing the droplet to enter the bottle beam. The increasing velocity following from photophoretic interactions is illustrated by the color code of the trajectories in Fig. 5 with respect to the color bars on the left side for *v*_*y*_ for the x/y-view and the right side *v*_*z*_ for the y/z-view of each subfigure of Fig. 5, respectively. The event of interaction is marked in (c), from entering the trap (1) through several interactions inside the bottle beam (2–4) until the droplet left the trap in z-direction with a very high velocity. Please note that *v*_y-terminal_ is the same for each droplet as far as they reach the bottle beam. Figure 6 shows the change of velocities for x- (*v*_*x*_), y- (*v*_*y*_) and z- (*v*_*z*_) directions, extracted from the videos in the case of Fig. 5(c) (see supplementary videos, frames 485–520). The numbered locations in (c) are connected with the corresponding time in Fig. 6. In both graphs, the oscillations in y and x are obvious and coincide with the observations in the videos. Additionaly *v*_*x*_ and *v*_*y*_ decrease with each interaction whereas *v*_*z*_ increases tremendously. The same conclusion follows from Fig. 7 were *v*_*x*_, *v*_*y*_ and *v*_*z*_ are given for the remaining subfigures (a), (b) and (d) of Fig. 5. In an analogous manner the interactions are marked and reveal several oscillations inside the bottle beam. The droplet related to Fig. 5(b) is an exception, since the droplet is too far away from the entry point and bounces off the outer wall. However, this single interaction leads to a strong acceleration in all three directions with the highest component in z-direction. This means that the axial movement of the droplet can not be stopped by the converging cone of conical refraction. In contrast, it has been demonstrated in previous experiments on solid absorbing particles that absorbing solid particles can be reliably trapped in the CR bottle beam^24^. The difference between trapping solids and liquids is due to specific droplet effects as convection, that require the necessary intensity gradient for an efficient interaction be higher than for solids^23^. Thus, it can be concluded, that a falling droplet of *v*_y-terminal_ ≈ −70 mms^−1^ can be reliably stopped by the intensity gradient in the focal plane. Yet, during the interaction, it is accelerated not only in the transverse, but even stronger in the axial direction. We were able to accurately resolve this motion. The side view images in Fig. 5 reveal that the axial end speed of the droplet *v*_*z*_, just before it leaves the field-of-view is as high as *v*_*z*_ ≈ 507 mms^−1^, is many times higher than its terminal falling velocity, and thus obviously too fast to be stopped by the blurred intensity gradient between the three “sharp” planes (compare Fig. 3). In order to balance the axial force, counter-propagating beams folded with two CR bottle beams can be implemented.

## Conclusion

We have demonstrated conical refraction optical bottle beams as optical traps for absorbing droplets. Its beam structure was characterized by an axial scan through the trapping volume which verifies the ability to open the top of the beam by adjusting the light polarisation. A sophisticated dual-view setup was developed, which allows the observation of the droplets from two perpendicular angles on the same camera chip. Thereby, a high 3D localization is possible without the need for additional timing adjustments. A partially opened optical bottle has been identified as being an ideal configuration that allows droplets to enter, yet needs no time-critical switching. The lateral confinement of absorbing droplets could be nicely visualized by multiple bounces of the droplet against the bottle beam walls. Ultimately, the strong axial force pushes the droplets out of the trap again by accelerating them up to *v*_*z*_ ≈ 507 mms^−1^. As a next step towards a 3D trap, we suggest the implementation of a counter-propagating CR bottle beam-based trap, which cancels strong axial acceleration. Due to the complex polarization structure of the CR ring, a combination of coherent and incoherent superposition and its influence on the flexibility to open and close the bottle need to be investigated.

## Electronic supplementary material

photophoretic droplets bottle beam
